# Implementing a free school-based fruit and vegetable programme: barriers and facilitators experienced by pupils, teachers and produce suppliers in the Boost study

**DOI:** 10.1186/1471-2458-14-146

**Published:** 2014-02-11

**Authors:** Anne Kristine Aarestrup, Rikke Krølner, Thea Suldrup Jørgensen, Alexandra Evans, Pernille Due, Tine Tjørnhøj-Thomsen

**Affiliations:** 1 Centre for Intervention Research in Health Promotion and Disease Prevention, National Institute of Public Health, University of Southern Denmark, Øster Farimagsgade 5A 2nd floor, 1353 Copenhagen K, Denmark; 2 Michael & Susan Dell Center for Healthy Living, The University of Texas School of Public Health, Austin Regional Campus, 1616 Guadalupe, Suite 6.300, Austin, TX 78701, USA

**Keywords:** Implementation, Process evaluation, Fruit and vegetables, School, Intervention, Adolescents

## Abstract

**Background:**

Multi-component interventions which combine educational and environmental strategies appear to be most effective in increasing fruit and vegetable (FV) intake in adolescents. However, multi-component interventions are complex to implement and often poorly implemented. Identification of barriers and facilitators for implementation is warranted to improve future interventions.

This study aimed to explore implementation of two intervention components which addressed availability and accessibility of FV in the multi-component, school-based Boost study which targeted FV intake among Danish 13-year-olds and to identify barriers and facilitators for implementation among pupils, teachers and FV suppliers.

**Methods:**

We conducted focus group interviews with 111 13-year-olds and 13 teachers, completed class observations at six schools, and conducted telephone interviews with all involved FV suppliers. Interviews were transcribed, coded and analysed using qualitative analytical procedures.

**Results:**

FV suppliers affected the implementation of the FV programme at schools and thereby pupils’ intake through their timing of delivery and through the quality, quantity and variety of the delivered FV. Teachers influenced the accessibility and appearance of FV by deciding if and when the pupils could eat FV and whether FV were cut up. Different aspects of time acted as barriers for teachers’ implementation of the FV programme: time spent on having a FV break during lessons, time needed to prepare FV and time spent on pupils’ misbehaviour and not being able to handle getting FV. Teacher timing of cutting up and serving FV could turn into a barrier for pupils FV intake due to enzymatic browning. The appearance of FV was important for pupils’ intake, especially for girls. FV that did not appeal to the pupils e.g. had turned brown after being cut up were thrown around as a part of a game by the pupils, especially boys. Girls appreciated the social dimension of eating FV together to a larger extent than boys.

**Conclusions:**

Limited time and pupils’ misbehaviour were barriers for teachers’ implementation. Establishing FV delivery to schools as a new routine challenged FV suppliers’ implementation. Food aesthetics were important for most pupils’ FV intake while the social dimension of eating FV together seemed more important to girls than boys.

**Trial registration:**

Current Controlled Trials ISRCTN11666034.

## Background

Low FV consumption is an important risk factor for the development of obesity, cardiovascular diseases and cancer [1,2]. Adolescents in western countries do not consume the recommended amount of fruit and vegetables (FV) [3,4]. Systematic reviews of intervention studies indicate that theory-based, multi-component interventions which combine educational and environmental strategies are most effective in increasing FV intake among children and adolescents [5-9]. Multi-component interventions are complex to implement [10,11] and are often poorly implemented [12-15]. Thorough process evaluation may increase our understanding of critical aspects of the implementation process and thereby improve the impact of future interventions in this field [10,16]. Qualitative methods can provide an in-depth understanding of peoples’ experiences, practices and social interaction and are therefore useful for examining different aspects of the implementation process [17,18]. Despite the growing awareness of the role that qualitative research can play in the evaluation of interventions [19,20], little qualitative research has been conducted to gain a better understanding of results from randomised controlled trials [19,21].

The importance of availability and accessibility of FV as a significant predictor of adolescents’ FV intake has been confirmed in previous studies [5-7,22-24]. However, few studies have been published on factors influencing implementation of school-based interventions addressing availability and accessibility in order to increase FV consumption among adolescents. Results from these studies have identified the following barriers for provision of FV in schools: *insufficient funding*[25], *timing of FV delivery from suppliers*[25-27], *quality and variety of FV delivered, wastage due to large amounts of delivered FV*[26], *insufficient numbers of volunteers*, *ineffective communication between families and schools*[25], and *limited time in the curriculum for implementing the FV intervention*[28]. Identified facilitators include: *sufficient funding*[26], *participation of the entire school*[26]*, pupils’ willingness to try new foods*[27], *teachers acting as role models*[25,26], and *teachers facilitating class discussion of the served FV*[25].

The aim of this study was to explore implementation and identify barriers and facilitators for implementation of two environmental strategies which addressed availability and accessibility of FV in schools in a Danish multi-component intervention as experienced by pupils, teachers and FV suppliers. Barriers for implementation refer to the obstacles faced by participants’ in implementing the components [29] while facilitators for implementation refer to factors promoting participants’ implementation. Identification of the barriers and facilitators can contribute to a contextual understanding of the pupils’, teachers’ and FV suppliers’ conditions, approaches and receptions when implementing a school-based intervention.

### The study context

In Danish schools there is no national provision of school meals. Pupils usually bring their own lunch bag or buy lunch in a school canteen if available [30]. The children often eat lunch together with their class mates in the classroom during a lunch period around noon.

#### The Boost study

The Boost study is a theory- and evidence-based multi-component intervention which combines environmental and educational strategies in schools, families and local communities to increase FV intake among 13-year-olds’ (year 7) [31]. The design, implementation and evaluation of the intervention were guided by the systematic planning tool ‘the Intervention Mapping protocol’ [32]. The programme theory which outlines how the Boost study was expected to impact the distal outcome (FV intake) through changes in proximal outcomes (determinants of FV intake) [31] (Aarestrup AK, Due P, Suldrup Jørgensen T, Krølner R: A six-step protocol to systematic process evaluation of multicomponent cluster-randomized health promoting interventions illustrated by the Boost study, submitted) was informed by systematic reviews of determinants of adolescents’ FV intake, reviews of intervention studies, theoretical behaviour change techniques, by input from a planning group and Boost’s international steering committee, and by developmental work via focus group discussions with year 7 pupils [31].

The intervention was tested in a cluster-randomised controlled study design with 20 intervention- and 20 control schools randomly selected from a random sample of ten municipalities in Denmark. The Boost study was implemented at intervention schools for nine months during the school year 2010/2011.

The Boost study consisted of five components: 1) *Daily provision of free FV,* 2) *A pleasant eating environment,* 3) Class-based curricular activities, 4) Parental involvement through newsletters and meetings at school, and 5) Information sheets to sports- and youth clubs. In the present study we examine the implementation of the two school environmental components: *Daily provision of free FV* and *A pleasant eating environment*.

##### Daily provision of free FV

**Figure 1:**
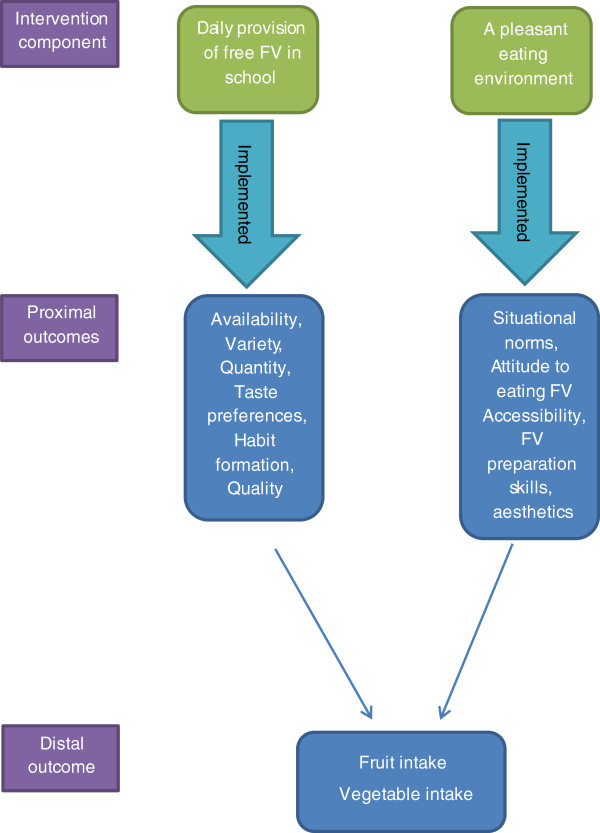
Boost Programme theory: Proximal outcomes of school environmental components.

This component was designed to increase daily availability of FV in the 20 intervention schools. On each school day throughout the intervention period all pupils in year 7 were provided with one piece of fruit or vegetable in class for free. By providing all pupils with FV (class-based programme) we intended to create a social norm “in year 7 all pupils eat FV”. A cooperative owner of a chain of supermarkets co-financed the free FV provision to pupils and decentralised the delivery of FV to supermarkets located nearby intervention schools. The arrangement involved 18 local FV suppliers as two suppliers delivered FV to two schools each. On a monthly basis, the Boost project group determined the type of FV to be delivered to schools. To expand taste preferences towards FV, pupils were exposed to a variety of FV during the intervention period. The suppliers were asked to deliver FV to schools in the morning twice a week. The proximal outcomes expected to be changed or developed by this intervention component are listed in Figure 1. The intervention component was based on the following theoretical behaviour change techniques: *facilitation, visual cues, taste acquisition theory* and *habit formation*[12,16,23,24,33-35].

**Figure 2:**
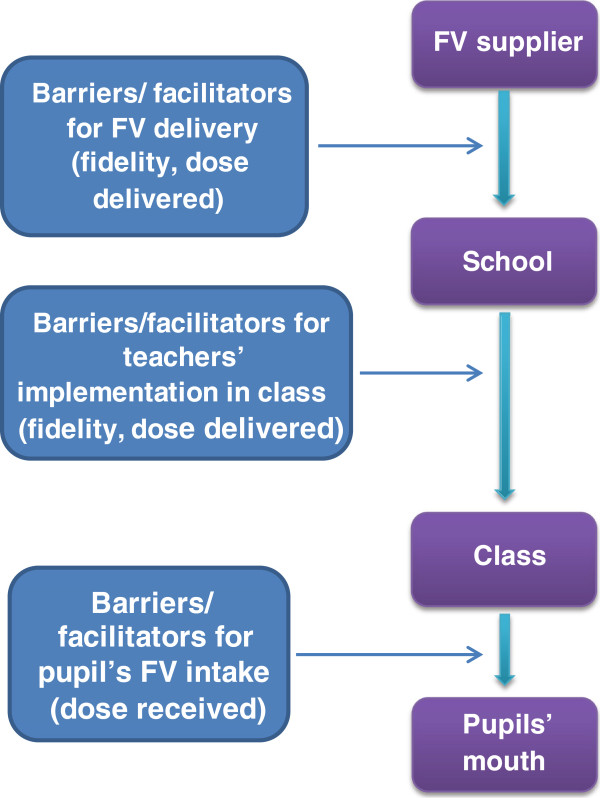
**Stages of implementation of *Daily provision of free FV *** **and potential points of barriers and facilitators.**

The process of implementing this component comprised of three stages: 1) from FV supplier to school, 2) from school to class, 3) from class to pupils’ mouths. We examined barriers and facilitators for implementation in all three stages (Figure 2).

##### A pleasant eating environment

This component was designed to support pupils’ intake of the delivered FV by 1) increasing accessibility of FV and 2) changing social and situational norms related to FV eating in school by creating a social practice in schools of pupils preparing and eating FV together in class. Teachers were encouraged to implement a daily FV break during a class lesson or a break where pupils could eat the provided FV together. The teachers were encouraged to designate pupils in each class as “FV hosts” to be responsible for bringing the FV to the classroom, cutting it up in appealing serving sizes, serving it to their classmates and cleaning afterwards. By this element of peer-led intervention, adolescents were involved actively in the implementation of the Boost study and learned FV preparation skills. This was also a strategy intended to help the teachers with the implementation. To increase multiple dimensions of accessibility e.g. making the FV appealing and convenient to eat, each class was provided with a class kit with tools for cutting up FV. This kit also contained lemon juice to sprinkle on cut FV to avoid enzymatic browning. The teachers were asked to eat FV with the pupils to act as positive role models (see Figure 1 for the proximal outcomes expected to be enhanced by this component). The theoretical behaviour change techniques underlying this component included *habit formation, reinforcement, social comparisons, peer modelling, facilitation and cues, skills training*[12,16,23,24,33-35].

At each intervention school, two teachers were appointed as coordinators of the intervention. Two teachers from each school, preferably the coordinators, were invited to a pre-intervention workshop to prepare for their role as implementers of the Boost study.

## Methods

The implementation of the multiple intervention components was examined by use of a systematic process evaluation protocol developed specifically for the Boost study (Aarestrup AK, Due P, Suldrup Jørgensen T, Krølner R: A six-step protocol to systematic process evaluation of multicomponent cluster-randomized health promoting interventions illustrated by the Boost study, submitted).

In this study, barriers and facilitators of three main process evaluation concepts were explored to identify conditions affecting implementation: 1) *dose delivered*: the extent to which the components were delivered to pupils; 2) *dose received*: the extent to which pupils received and ate the delivered FV and 3) *fidelity:* the extent to which participants’ implementation of the components adhered to the implementation protocol e.g. a written folder for teachers on how to promote a pleasant eating environment. In addition, we explored a fourth key process evaluation concept: *reach,* defined as dose received by subgroups to explore if the reception of the intervention differed by gender [16,36].

Focus group interviews with pupils and teachers, observations of FV breaks and telephone interviews with suppliers were conducted. Schools were selected for focus group interviews and observations in order to obtain maximum variation [17] in geographical location and in teachers’ satisfaction level with the Boost FV delivery at the beginning of the intervention period.

Semi-structured interview guides for pupils, teachers and suppliers and an observation check list were developed specifically for the Boost study, guided by the Boost process evaluation protocol and inspired by tools applied in the HEIA study [33] and Pro Children study [34] (see Additional file 1, Additional file 2, Additional file 3, Additional file 4).

**Figure 3:**
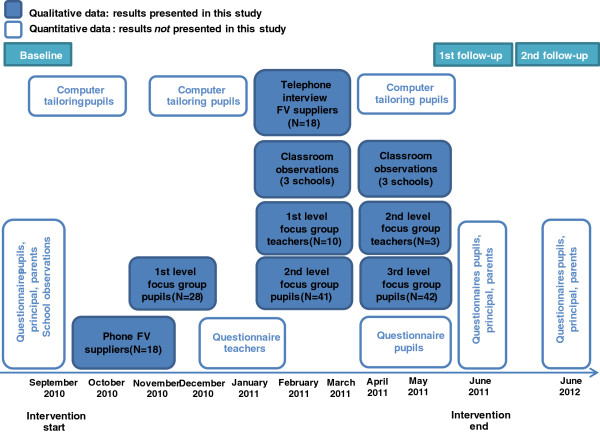
**Timeline and data collection methods used for the process and effect evaluation of two components in the Boost study: *Daily provision of free FV *** **and *A pleasant eating environment*** ***.***

Figure 3 shows the time line of the data collection for the entire Boost study.

### Focus group interviews with pupils and teachers

We chose the focus group interview format to 1) hear pupils’ and teachers’ experiences with the FV programme, 2) allow participants to challenge and reflect on each other’s perspectives, and 3) to observe participants’ interactions throughout the interviews [37,38]. The interviews focused on participants’ experiences with and views on the class-based Boost FV programme (see Additional file 1, Additional file 2).

AKA conducted 16 focus group interviews with a total of 111 pupils at eight schools. Each focus group consisted of 6-7 pupils selected by the teachers. We conducted both gender homogenous and gender heterogeneous groups to capture potential different dynamics deriving from different focus group compositions.

AKA conducted three focus group interviews with teachers at three schools, two single interviews with teachers at one school and one single interview at another school, in total 13 teachers. These interviews were mainly conducted at the same schools as the pupil focus groups.

Focus group interviews with pupils and teachers took place at three and two different time points, respectively (Figure 3). This approach was chosen to 1) illuminate potential variations in dose delivered and dose received during the intervention year and associated barriers and facilitators and 2) to refine the interview guides according to experiences and information identified in the first rounds [18]. For example, the first round of interviews revealed that pupils threw with the FV and that this acted as a barrier for teachers’ implementation of the FV programme (dose delivered) and for pupils’ intake of FV (dose received). We therefore added questions concerning this behaviour to the interview guides for the next rounds of interviews.

We aimed at including both Boost coordinators and regular teachers in the interviews as well as pupils from both coordinators’ and regular teachers’ classes. This approach was chosen as coordinators’ and regular teachers’ views, experiences and involvement in the intervention could differ according to feeling of responsibility and interest and influence pupils’ attitude towards Boost.

A co-moderator with detailed knowledge about the Boost study assisted AKA during the interviews and took notes and posed additional questions if relevant. All interviews were conducted at schools and lasted approximately 45 minutes. Interviews were audiotaped and transcribed verbatim by AKA or colleagues affiliated with the Boost study.

### Observations of the FV break

Observations are well suited to investigate both relations between people and relations between people and their surroundings [17,18,39]. AKA and two colleagues from the project group observed the implementation of the FV break at six schools in order to document the dynamics and atmosphere related to the FV break among pupils and teachers and to explore different implementation practices across school classes, teachers and pupils (see Additional file 3).

At second and third focus group visits, observations were conducted (Figure 3) to be able to observe implementation practices at different time points. During the focus group interviews, we asked pupils and teachers to elaborate on important findings from the observations.

### Telephone interviews with FV suppliers

We conducted telephone interviews (lasting approximately 20 minutes) with all 18 suppliers about their reasons for participating in the Boost project and their experiences with the FV delivery to schools (see Additional file 4). The interviews were audio recorded and notes were taken.

### Ethical considerations

The Boost study adheres to all Danish ethical standards and has been approved by the Danish data protection agency (J.nr. 2010-54-0974). Prior to participation in the Boost study, the school boards, parent committees and pupil committees were informed that the pupils would be interviewed about their experiences with the Boost study. Parents who did not want their children to participate in the evaluation of the Boost study could indicate this when completing the parental baseline and follow-up questionnaires (passive consent). Furthermore, the voluntariness and anonymity of participation was emphasised to pupils, teachers and suppliers at the beginning of each interview. No personal identifiers were included in the transcripts.

### Data analysis

AKA performed a thematic analysis of the interviews. The qualitative data analysis was both an inductive and deductive process [17,18]. We let the material talk (data-based coding), but we also sought answers to the specified research questions regarding implementation and barriers and facilitators for implementation (theory-based coding).

The recorded focus group interviews with pupils were listened through and the transcripts were reviewed. First, we performed a within-case analysis of each interview and coded the transcribed interview into categories (e.g. *Cut up FV*). Secondly cross-case analyses were conducted to identify similarities and differences across interviews and categories were gathered into overall themes (e.g. *Perceived FV accessibility*). Each theme was categorised as a barrier or a facilitator for the listed process evaluation concepts e.g. fidelity or dose delivered (Aarestrup AK, Due P, Suldrup Jørgensen T, Krølner R: A six-step protocol to systematic process evaluation of multicomponent cluster-randomized health promoting interventions illustrated by the Boost study, submitted). Quotes were drawn from the interview material to illustrate the findings [18].

The themes identified in the pupil interviews guided the initial identification of themes in both the transcribed teacher focus group interviews and the notes from interviews with suppliers. New themes of importance for teachers’ and suppliers’ implementation were also allowed to emerge.

The coding and interpretation of results were continuously discussed with co-authors and researchers outside the Boost project group (investigator triangulation) [18].

## Results

Below, the barriers and facilitators are presented according to selected proximal outcomes of the two studied components: availability (*Daily provision of free FV)* and accessibility and social norms (*A pleasant eating environment)*. These proximal outcomes were selected based on the most recurrent findings. The results identify which conditions that comprise a barrier or facilitator for fidelity, dose delivered and dose received of the intervention. Furthermore, variation in implementation of the FV programme is presented.

**Figure 4:**
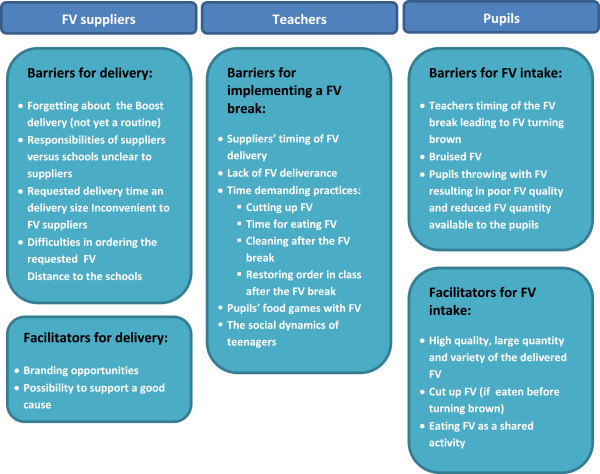
Barriers and facilitators for implementation of the Boost FV programme experienced by FV suppliers, teachers and pupils.

In Figure 4 the identified barriers and facilitators for FV suppliers’, teachers’ and pupils’ implementation are summarized.

### Availability: delivery and timing

The suppliers’ timing of FV delivery was identified as a barrier for teachers’ implementation and the pupils’ intake of FV in both pupil and teacher interviews. At some schools, the suppliers at times delivered the FV too late in the day to fit the class schedule. At the beginning of the intervention period some schools did not receive FV at all as some suppliers did not know they were supposed to deliver the FV to schools and therefore expected the teachers to pick up the packed FV themselves. These problems caused frustrations and disappointment and affected the participants’ attitude towards the Boost study.

Teacher: It was very frustrating as we had promoted it in class and to the parents. That now we were starting this Boost project and there would be fruit every day. And then I have 28 pupils and at least 56 parents who don’t understand where the fruit is.

Observations confirmed the problem. The delivery problems diminished after the first 2-3 months of the intervention period.

Interviews with suppliers revealed that sometimes they forgot to deliver the FV to the schools because it was a new task.

FV supplier: The hardest part is to remember to deliver the fruit and vegetables [to the schools]. It is a new routine that has to be integrated.

Other barriers for suppliers’ timely delivery to schools included lack of communication between personnel within the supermarket, and mismatch between the schools’ requested time for delivery and the suppliers’ schedule. Some suppliers were not able to deliver certain FV as the FV were either out of season and therefore difficult to order, or because the suppliers ran a small-scale supermarket with a limited assortment. The delivery size of FV to schools was not perceived as convenient by all suppliers as they were forced to repack their regular FV boxes. Delivery to schools located far away was perceived as an inconvenient and non-appealing task by some suppliers. However, the majority of suppliers did not experience the Boost delivery as time consuming. Their participation and implementation was facilitated by the prospects of publicity and branding, the opportunity to show goodwill to the cooperative owner, the school, pupils and parents and to support a good cause.

FV supplier: It is an advantage that the parents are aware that their children get fruit from our supermarket. It creates goodwill.

In summary, the timing of suppliers’ FV delivery or their lack of delivery acted as barriers for teachers’ implementation and pupils’ dose received and compromised intervention fidelity in the first months of the intervention year. Dose delivered by suppliers was challenged by barriers such as suppliers forgetting the order, miscommunication, mismatch between the requested time of delivery to schools and the supermarkets time schedule, difficulties of ordering the specific FV, inconvenient delivery size and distance to the school. The possibility of branding acted as a facilitator for suppliers’ implementation.

### Accessibility: food aesthetics, convenience and teachers’ timing

Pupils and teachers identified - in agreement with the programme theory of Boost (Figure 1) - high quality of FV, and large quantity and variety of the delivered FV as facilitators for pupils’ intake. These dimensions were influenced and to a large extent determined by the FV suppliers. Most pupils experienced and appreciated that abundant FV were delivered. Variety of the FV delivered enhanced the pupils’ use of the FV programme as they found it boring if they were to eat the same FV every day.

Pupils expressed that the accessibility and appearance of the FV highly influenced their intake. Most pupils preferred to eat FV cut up as snacks as they found it more appetising, easier to eat, and cosier (see for explanation of the notion cosy inserted below).

Cosy: In this article we use the term cosy to refer to the Danish phenomenon ‘*hygge*’. *Hygge* is a social phenomenon which in Denmark has an almost iconic status in representing a style of being together. The concept embraces a certain quality of sociality and signifies a safe, low-key, intimate form of socialisation, where the closeness is often based on sharing food or drinks. *Hygge* is a ubiquitous concept in the Danish culture and hard to translate into other languages [40]. The pleasant eating environment component was designed to promote *hygge* around FV eating in class.

Girl: Well it is like cut up and so, just ready to eat, really delicious.

Girl: I never eat fruit at home because I cannot bother to take an apple and eat it. I find it boring.

Girl: You see, nobody bothers cutting FV up at home. Parents, you see, do not cut up an apple for you to eat.

Furthermore, contrary to being provided with one piece of fruit or vegetable, the cut-up FV enabled the pupils to taste different FV. Despite the fact that the same number of FV were delivered in class each day, the pupils felt they received a larger amount of FV when it was cut up compared to when they received one piece each and they liked that feeling. However, some pupils also perceived this as a drawback because it became more difficult to share the FV evenly and involved more counting of pieces and pupils arguing in class.

Several teachers also experienced that cut-up FV were popular among pupils and led to higher intake, but the practice was considered to be very time consuming.

Teacher: At the beginning I found the FV programme troublesome, I have to say. I think it took a lot of time, so I have tended to: –“if you want an apple, you take a whole apple”. I refuse to slice them all into ten pieces. It takes too long [time].

The teachers influenced dose received as they decided when the pupils could eat FV. Some teachers allowed pupils to eat FV whenever they wanted during lessons, while others were more restrictive.

According to pupils, some teachers did not allow them to eat FV during lessons because it would take time from the teaching, would leave a mess or create chaos in class.

Time was identified as the main barrier for teachers’ implementation of the two intervention components. Several of the involved had taken over a new class at the beginning of the intervention year and therefore lacked energy to engage in the different components of the Boost study. Time consuming practicalities related specifically to the FV programme included: cutting up FV (both when performed by the teacher and the pupils), restoring order after the pupils’ eating FV in class, cleaning, pupils’ food games with FV, and allocating time for eating FV.

The teachers’ control of the timing for eating FV affected not only pupils’ access to FV but also the appeal of the FV delivered: Sometimes the FV were cut up at the break prior to the class lesson, but the pupils were not allowed to eat it until the end of the lesson or after the lesson. This caused enzymatic browning of the FV.

Boy: If we have S [teacher] then it [FV] is just lying there and is slowly turning brown before we are allowed to eat it.

This timing issue limited pupils’ interest in eating the FV as they perceived it as brown, gross, unappetising, greasy or dry. According to the interviewed teachers and pupils, the lemon juice which was provided in the class kit to prevent browning was only used a few times in the beginning of the intervention period or not at all.

Pupils would like to eat the FV as soon as it was brought to the classroom but their request was handled differently by teachers.

Girl: D [teacher], she is okay. When we ask for it [FV] we can have it, but T [teacher], he does not allow us to have it.

Girl: If we ask for it, he just says ’ah, but now you will not get it for sure’.

Bruised FV were rejected and described by the pupils as ‘disgusting’ and some pupils associated brown with putrefaction.

Girl: Well, I think apples and pears when they become brown then they are not… There is nothing wrong with it, but you do not really feel like eating it because it does not look that delicious.

Girl: At the end, it is getting all greasy on the surface

Interviewer: Does it mean that you do not eat the entire fruit or just not that part?

Girl: I do not eat it.

Girl: I do not eat it if it is like that all over.

Girl: The majority does not eat it.

Girl: The boys might eat it, but the girls do not.

The interviews indicated that appearance of FV was more important for girls’ intake compared to boys.

The teachers confirmed the importance of appearance for pupils’ intake.

Teacher: They do not eat it if it is the least bruised or the least something. It has to look very, very perfect otherwise…

Teacher: Well, if we cut it [FV] up and it turns brown then they will not eat it, so you have to be rather fast [distributing FV to pupils].

We also experienced pupils’ negative attitude towards browned fruit during the observations. In one class, the pupil who was FV host highlighted that it was a good idea to cut off brown areas before serving fruit to classmates.

Furthermore, if the appearance of the FV did not appeal to the pupils or did not match the pupils’ taste preferences or when the pupils were bored, they sometimes used the FV for a different purpose. In all interviewed classes, pupils, especially boys, were throwing with the FV to some extent. The FV were thus transformed into inedible objects to throw and play with.

Boy: But some of the fruit, it just make you, throwing, you can throw really well with it.

Boy: Such a tomato, it just lays well in the hand.

Boy: Or those radishes, well radishes, nobody likes radishes anyway.

Boy: We throw them. If there is something we do not like, then we throw it.

Some pupils, mostly girls, found the food games annoying as it resulted in 1) a smaller quantity of FV being available to the class, 2) threats from teachers to end the FV programme, and 3) a dirty classroom with FV all over the place.

Girl: There is hardly anyone who will eat oranges and clementines because the boys are throwing with them and you do not know if they have thrown it and put it back. And then when you open it, it is all gross.

The food games challenged the intention of creating a pleasant eating environment.

We also experienced the practice of playing and throwing with FV during observations at one school. During the observation, the FV were thrown in a game where boys and girls teased each other or flirted. The game took place so everyone could see and hear it and the involved pupils’ seemed to find it cool.

The teachers found the throwing of FV frustrating and draining.

Teacher: The children have not been able to behave properly with regards to throwing the FV. Honestly, sometimes it was so disturbing. If you saw how the classroom looked, tomato juice was running down from ceilings and walls.

According to many teachers, the FV programme required more teacher control than initially expected. Because the pupils in many classes could not administer the FV break by themselves, several teachers ended up allocating time for the FV eating during lessons.

Teacher: We have been forced to lock the classroom during breaks and to serve the fruit during lessons. It [the Boost FV programme] has become more teacher-guided than intended. That took a lot of energy.

Some teachers mentioned that they could not let the pupils do something together while eating FV during lessons, as the pupils could not control such a space.

Teacher: Sometimes you have to run the classes so strictly that the pupils are about to become suffocated. They can’t handle it if there is no structure. That is why you need to schedule it, now we do this and now we do that, the fruit is here. The prospect of a break and it is exploding.

The observations revealed rather different ways of implementing the FV break in different classes. Some teachers allowed pupils to cut up FV for themselves whenever they felt like it, both during lessons and breaks. Other teachers locked up the cutting tools and held the FV break at a prescheduled time.

Observations and interviews indicated that pupils in some classes had learned to administer the FV host responsibility of cutting up the FV without much teacher guidance. The throwing of FV also seemed to have declined by the end of the intervention period.

Most of the interviewed teachers referred to year 7 pupils as a special age group challenging implementation. According to the teachers many things are going on in this age group due to psychological, physical and social developmental changes and the pupils need a lot of structure.

Teacher: Ah well it is just that, well it is year 7 and everything is running around their heads.

Teacher: Well you know, there is really nothing getting through to them, they are “under reconstruction” those boys at year 7. They are simply all gone.

In summary, high quality, quantity and variety of FV were facilitators for dose received. As expected according to our programme theory, accessibility and appearance acted as both facilitators and barriers for dose received and were influenced by the teachers’ timing of the FV break. Teachers’ lack of time was a barrier for dose delivered and fidelity. The throwing of FV had an impact on the implementation by affecting 1) fidelity and dose delivered of the pleasant eating environment component as some teachers made more restrictions resulting in less pupil involvement and 2) dose received of the free FV as the pupils rejected the thrown FV. The teachers perceived the pupils’ age and social dynamics in the pupil group as barriers to teacher dose delivered and pupils’ dose received of the intervention.

### Social norms: eating together

The interviews illustrated that pupils who shared and ate the same FV together formed a sense of community around the FV programme.

Interviewer: What makes it cosy, do you think?

Girl: Maybe the fact that we do the same thing and that it is something we do together.

Girl: Also we can sit and talk.

Girl: Yes, so we are a bit united in what we are doing.

Interviewer: Would it be different if you ate fruit that you had brought from home?

Girls: Yes.

Interviewer: How is it different?

Girl: Ah well, it is not served, you have to take it out of your bag yourself and things like that.

Girl: And then we do not bring the same fruit.

Interviewer: So it makes a difference that everyone has the same fruit.

Girl: And the same fruit, so you can talk about it.

The pupils appreciated that the FV programme was for everyone and some pupils expressed that it became a habit to eat FV in class and that they affected each other’s eating habits. Especially girls highlighted the cosiness of sharing the FV and being allowed to chat with each other while eating. Some girls perceived the implementation of the FV programme as getting an extra break, making school work in class less boring.

Girl: Well, we eat it together and then sometimes we are allowed to talk a bit when we are not working on something. It is almost like free time or freedom when we eat it.

The importance of having this free time, a specific time that belonged to the pupils, was also illustrated in an interview with a group of girls expressing their strong discontent with being forced to use their break to cut up the FV.

Contrary to the girls, the boys did not experience the FV eating as a shared activity.

Interviewer: How do you like eating FV together with your classmates?

Boy: It is okay. I do not really think about that we are eating it together as you just take some and then eat it by yourself.

Interviewer: So you do not experience it as a shared activity as such?

Boy: No, not at all.

These boys’ intake was not motivated by eating FV at the same time as classmates. Some boys felt that the girls appreciated the social aspects of eating FV together to a greater extent than them. Our interview questions prompted some boys to reflect on the eating situation and they concluded that they actually talked more with each other while eating the FV than while eating their packed lunch. During our observations we experienced that boys left the classroom as soon as the break began e.g. to go outside to play football while several girls stayed in the classroom and ate FV. In some interviews boys expressed their discontent with not getting FV when they returned from playing football as it had already been eaten.

The teachers reported that pupils enjoyed having FV in class during lessons and/or breaks. The teachers were unsure whether the pupils experienced the FV programme as creating a sense of community. According to some teachers, their pupils distinguished themselves from pupils from other classes by having the privilege of free FV. They liked to point out that the FV belonged to them. In some classes, our observations showed that pupils were eating the FV during breaks in smaller groups while talking. In other classes little social interaction took place as the pupils ate FV in the lesson while working.

In summary, eating FV as a shared activity acted as a facilitator for dose received by the pupils, especially among girls. The interviews indicated a gender differential appeal and reach of the social aspects of the pleasant eating environment component. The pupils’ sense of being unique because they were chosen to get a FV programme might also act as a facilitator for dose received. Lack of time served as a barrier for teachers’ delivery of *A pleasant eating environment* and their fidelity to this component.

## Discussion

We begin this section by summarizing the main findings of identified barriers and facilitators for implementation. Secondly, we discuss the main findings according to the programme theory and specific proximal outcomes the intervention was designed to address. Lastly we address implications for research and practice and the strengths and limitations of the study.

Teachers controlled the implementation of the pleasant eating environment component by deciding if the pupils could eat FV during their lessons and whether it was cut up. Teachers implemented the FV programme differently e.g. some teachers cut up the delivered FV while others did not. Time was an important barrier for teachers’ implementation of the FV programme both in terms of teachers not wanting to spend time on preparing the FV, to allocate time for a FV break nor to spend time on controlling the pupils who were unable to administer a FV break. Other studies also highlight time issues as crucial for implementation of interventions [6,14,28,41-45] (Suldrup Jørgensen T, Krølner R, Tjørnhøj-Thomsen T, Aarestrup AK, Due P, Rasmussen M: Barriers and facilitators for teachers' implementation of the curricular component of the Boost intervention targeting adolescents' fruit and vegetable intake, submitted). A different aspect of time - not mentioned in previous studies - was identified as a barrier in our study: Teachers’ timing of the FV break and the cutting up of the FV turned out to be a barrier to the pupils FV intake as it affected food aesthetics. This illustrates a conflict of interest between pupils and teachers that can hinder adequate implementation. Several teachers preferred the preparation and eating of FV to take place during breaks, but in several classes the pupils could not administer this. Pupils’ eating of the delivered FV was compromised by supplier’s timing of FV delivery, and the quality and variety of the delivered FV. These findings are supported by previous studies [26,27]. The pupils appreciated having a FV break as they experienced the time as their own time and they felt that it provided them with a sort of *freedom* and *free time*. At the same time some pupils expressed a discontent with spending their breaks on preparing the FV.

### Programme theory revisited

Increased knowledge about factors affecting implementation is important to understand whether an eventual lack of intervention effect is due to implementation failure or a badly designed intervention [10]. This study addressed the assumptions of the Boost programme theory and selected proximal outcomes of the two components (Figure 1) which is an important part of process evaluation [46].

To increase adolescents’ intake of FV, the free FV programme and the pleasant eating environment components were designed to address large variety, high accessibility, sufficient quantity, quality, appearance of FV and social norms related to eating FV. The qualitative interviews confirmed that these factors enhanced adolescents’ FV intake (dose received). However, our findings indicate that success in changing these proximal outcomes of the intervention components could be challenged by inadequate implementation [16] such as suppliers’ timing of the FV delivery, teachers’ timing of FV eating, teachers’ priority of conducting a FV break and pupils not being allowed to cut up FV. Our findings underscore it can be challenging for teenagers, especially boys to handle a break involving eating FV together (one of the dimensions of the component *A pleasant eating environment*).

In agreement with our programme theory and other studies, we found that cutting up FV into snacks promoted FV intake among adolescents [22,31]. However, cutting up FV was not sufficient to ensure intake as it potentially collided with food aesthetics/appearance which is another important determinant for children’s intake of FV. Several studies support the importance of food aesthetics in children's choice of FV [47]. Carlsen et al. highlight food aesthetics and the context as influencing what people wish to eat [48].

In our study, the notion of cosiness and gender played a significant role for the pupils’ intake of FV. The pupils described the aspect of eating *the same* FV *together* as cosy and something that united them. Eating the same FV was important because they could talk about what they were eating. The finding of the importance of eating together supports our programme theory. Simmel describes communal eating and drinking as unleashing an immense socialising power [49]. Makela (2009) also points to the social aspect of eating [50]. According to Makela (2009), the notion of eating as reproduction of social relations is crystallised in meals that allow people to eat the same food at the same time and therefore to share the ideas of commensality attached to meals [50]. Our findings indicated that it can be difficult for pupils in this age group to articulate the sociality of eating when asked about it in interviews. Similarly, Ross reported that pupils aged 10-12 years had difficulties in identifying the importance of eating the same [51].

The findings from our study indicate that the social aspect of eating together appealed more to girls than to boys, which is supported by another study [51]. This may compromise the impact of the intervention among boys. The throwing with FV among pupils was an unforeseen side effect of increasing pupils’ access to FV. This threatened our intentions to create a pleasant environment for eating FV and conflicted with teachers’ interests. The pupils invented their own social aspect of the FV programme through the FV games. Other studies have not reported this side effect of providing pupils FV. The obtained knowledge on the implementation challenges may provide an input to adjustment of the programme theory e.g. through introducing a gender differentiation of the intervention.

### Implications for research and practice

The appearance and aesthetics of the FV are important dimensions to consider when developing an intervention to increase pupils’ FV intake and even more important when attempting to reach girls. The findings from this study suggest that the concept of FV accessibility should be revised: It is not sufficient to make FV accessible to pupils by cutting it up. The timing of the FV preparation is crucial in order to prevent the FV from turning brown before the pupils can eat it. From this qualitative implementation study we learned that a greater focus should be put on securing the teachers’ fidelity to implementation of the intervention components. The enzymatic browning of FV could have been prevented if teachers had used lemon juice as suggested by the project group or if they had scheduled the FV preparation, so the FV could be served immediately after being cut up.

In the Boost study we attempted to account for teachers’ busy schedule by encouraging teachers to delegate the FV preparation tasks to pupils. In spite of the designation of FV hosts among pupils, our findings show that teachers still spent much time on the implementation. Future studies could profit from exploring and taking the reality of teachers’ limited time thoroughly into account e.g. by providing more detailed and time saving guidelines on how to implement a FV programme. Another way to facilitate teachers’ implementation of the FV programme would be that politicians made FV breaks a compulsory part of the school curriculum.

Our study highlights the importance of including a social aspect in dietary interventions, especially to target girls’ eating habits. We intended to reach boys and girls equally by the intervention [31], but we may have increased the gender inequality in FV intake [52,53] as this part of the pleasant eating environment component appealed more to girls. This hypothesis can be tested in the quantitative effect analysis of the Boost intervention. A different approach may be needed to appeal to and reach boys for example integrating FV intake with physical oriented activities such as playing soccer in the class breaks [51]. An exploratory qualitative study of what it means to be a year 7 pupil and the social and gendered dynamics of this age group may provide information about their preparedness for receiving an intervention like Boost. Furthermore such a study may provide insight and relevant knowledge for development and implementation of future interventions e.g. by conducting participant observations throughout a longer period.

Our findings indicate that the introduction of a FV programme to this age group requires a running-in period of the intervention before it runs efficiently.

Furthermore, the identified FV delivery problems show that establishing new procedures for suppliers might take some time before being integrated as a daily routine. This calls for a longer intervention period. Securing incentives such as branding facilitate suppliers’ participation.

### Study strengths and limitations

Teachers recruited pupils for focus groups which may have resulted in the participation of more skilled and socially advantaged pupils potentially giving a more positive picture of the implementation process and of pupils’ reception of the intervention (selection bias). On the other hand, this recruitment strategy may have provided us with richer data as teachers most likely included pupils they knew would be able to contribute constructively to a focus group discussion.

We aimed to include both coordinators and regular teachers in the focus group interviews but in some schools it was only possible to involve coordinators which again may have resulted in a more positive account of the implementation process as they 1) knew most about the project 2) were more engaged due to their coordinator responsibility and 3) maybe had a more positive attitude.

To limit social desirability bias in pupils’ and teachers answers we began the focus group interviews by clarifying that we were interested in both their positive and negative experiences with the FV programme. We did not perceive that teachers or pupils withheld any information during the interviews. In a few interviews we had to remind the pupils that we would not inform their teachers of their answers. This was mainly in relation to their representation of the food games. The suppliers were more reluctant to share their views and did not go much into details. Thus, we may not have received a comprehensive picture of their participation.

Scholars discuss the importance of separating the role of intervention deliverer and evaluator when conducting evaluations due to objectivity issues [54]. In this study, we perceive it as an advantage that AKA, a member of the project group conducted all interviews. AKA’s thorough inside knowledge of the intervention made it easier to ask more detailed questions related to implementation. We did separate the role of deliverer and evaluator in the sense that AKA was not the one handling the project group contact with the teachers in relation to the FV delivery during the intervention period.

The collection of data at different time points throughout the implementation period adds the strength of identifying changes in the level of implementation. Our study identified that the intervention required a running-in period before it was delivered properly.

Data source triangulation is a merit of this study. If we had not interviewed both teachers and pupils we would not have obtained the detailed information on how teachers’ timing of serving cut up FV played a crucial role for whether pupils wanted to eat the delivered FV.

The different compositions of the pupil focus groups provided different group dynamics and knowledge. As an example, boy groups tended to put more focus on the entertaining dimension of playing with FV, while girl groups tended to comment on the games as annoying and disgusting. In one gender heterogeneous group we experienced that the FV games were also represented as a part of a game going on between boys and girls teasing each other.

The Boost process evaluation protocol (Aarestrup AK, Due P, Suldrup Jørgensen T, Krølner R: A six-step protocol to systematic process evaluation of multicomponent cluster-randomized health promoting interventions illustrated by the Boost study, submitted) was useful in securing the collection of relevant qualitative data for exploring the implementation process. Using different qualitative data collection methods adds to the strength of the study [17,18,55]. The focus group interviews contributed with valuable information through the interaction between the pupils, capturing for example the importance of the FV appearance. The observations illustrated how the social practice of eating FV together took place and differed between boys and girls and between different classes. Future intervention research could benefit from the strengths of integrating qualitative research methods thoroughly and early in the development of the intervention since they can provide an insight into the local context of the intervention and the perspectives of the participants.

## Conclusion

This study provides new insights regarding implementation of two environmental strategies to increase adolescents’ FV intake in the Boost study and factors affecting the implementation. The teachers implemented the FV programme differently. Securing high accessibility by cutting up FV may compromise another important facilitator for adolescent FV intake, namely appearance of FV. The aesthetics play an important role in getting adolescents to eat FV, why it is important to take this dimension carefully into account when attempting to affect their intake. The study also points to the importance of taking gender into consideration when trying to affect adolescents’ FV intake. Boys and girls value the social aspects of a class-based programme differently. Different time concerns of teachers such as a tight schedule and pupils’ lack of capability to handle the FV break by themselves influence teachers’ implementation of the FV programme. Suppliers’ coordination, a slow integration of a new routine and timing of the FV delivery challenge their implementation. The use of qualitative research methods is crucial for understanding the implementation process and context and participants’ perspectives on the intervention and should be included in all school-randomised controlled trials.

## Competing interests

The Boost study is part of Centre for Intervention Research in Health Promotion and Disease Prevention, National Institute of Public Health, University of Southern Denmark, Øster Farimagsgade 5A, 1353 Copenhagen K, Denmark. The Centre is funded by TrygFonden and the Danish Cancer Society. The Boost study is funded by a 5-year donation from TrygFonden including funding of each author and coverage of expenses related to intervention, implementation and evaluation. PhD scholarships for AKA and TSJ are co-financed by University of Southern Denmark. The free FV school programme in the intervention was co-financed by 1) FDB (a Danish membership organisation which owns Coop, a Danish chain of grocery shops) and 2) the EU School Fruit Scheme through the Danish Food Industry Agency, the Danish Ministry of Food, Agriculture and Fisheries. Copenhagen Food House contributed financially to the development of the teaching material. AE was funded by the Michael & Susan Dell Center of Healthy Living at the University of Texas, School of Public Health in Austin, Texas.

## Authors’ contributions

AKA participated in the design of the Boost study, conceived of the present study, performed the interviews and observations, analysed data and drafted the manuscript. RK participated in the design of the Boost study, conceived of the present study, contributed to interpretation of data and revised the manuscript critically. RK is the principal investigator of the Boost study. TSJ participated in the design of the Boost study and revised the manuscript critically. AE revised the manuscript critically. PD participated in the design of the Boost study, conceived of the present study and revised the manuscript critically. TT conceived of the present study, contributed to the interpretation of data and revised the manuscript critically. All authors approve of the final version of the manuscript.

## Pre-publication history

The pre-publication history for this paper can be accessed here:

http://www.biomedcentral.com/1471-2458/14/146/prepub

## Supplementary Material

Additional file 1 Topic areas for pupil focus group interview on the Boost fruit and vegetable (FV) programme. Click here for file

Additional file 2 Topic areas for teachers’ focus group interview on the Boost fruit and vegetables (FV) programme. Click here for file

Additional file 3 Observation guide for the Boost fruit and vegetables (FV) break. Click here for file

Additional file 4 Topic areas for interviews with fruit and vegetable (FV) suppliers on the Boost FV programme. Click here for file
