# Supplementary material for: Implementing a free school-based fruit and vegetable programme: barriers and facilitators experienced by pupils, teachers and produce suppliers in the Boost study
Source: BMC Public Health. 2014 Feb 11;14:146. doi: 10.1186/1471-2458-14-146 (PMC3946026; doi:10.1186/1471-2458-14-146)
Supplement: Additional file 1 — Topic areas for pupil focus group interview on the Boost fruit and vegetable (FV) programme. [file 1471-2458-14-146-S1.docx]

**Additional file 1. Topic areas for pupil focus group interview on the Boost fruit and vegetable (FV) programme**

1. Do you bring FV to school from home and have you changed that practice after the Boost FV programme has been introduced in your class?

2. How does the Boost FV programme take place in your class?

a) Who is bringing the delivered FV to the class?

b) Where are the FV stored?

c) Preparation of the FV: – who?, when?, Are FV being cut up?

d) How is the FV eating taking place – what are you doing while eating FV, a specific break where the class do something together?

e) When are you eating the delivered FV – during a lesson or a break, can you decide the time?

f) How much FV do you eat in class?

g) Who (e.g. girls/boys) eat FV?

3. How do you like the Boost FV programme?

a) Is the FV fresh?

b) How does the FV look and is the appearance of importance to you?

c) Is there delivered enough FV for all pupils?

d) Have you tasted new FV as part of the programme and do you like to taste new FV?

e) Do you prefer FV to be cut up or not?

f) What is good and what is bad about having a FV programme?

g) Do you eat more or less FV in total after the introduction of the Boost FV programme?

h) How do you think it will affect you when you do not have a FV programme next year?

4. How do you like eating the FV together with your class mates?

5. How has teachers’ involvement been in the Boost FV programme?

a) Is it the same teacher/teachers you eat the FV with?

b) Do all teachers allow you to eat the FV in their lessons?

c) What do your teachers think about the FV programme?

6. Do your parents appreciate that you have a FV programme in school?
