# Supplementary material for: Implementing a free school-based fruit and vegetable programme: barriers and facilitators experienced by pupils, teachers and produce suppliers in the Boost study
Source: BMC Public Health. 2014 Feb 11;14:146. doi: 10.1186/1471-2458-14-146 (PMC3946026; doi:10.1186/1471-2458-14-146)
Supplement: Additional file 2 — Topic areas for teachers’ focus group interview on the Boost fruit and vegetables (FV) programme. [file 1471-2458-14-146-S2.docx]

**Additional file 2. Topic areas for teachers’ focus group interview on the Boost fruit and vegetables (FV) programme**

1. How have do you experienced the Boost FV delivery?

a) Has the FV been delivered at the time you needed it?

b) How is the quality, quantity and variety of the delivered FV?

c) How often do you communicate with the FV supplier and have you been content with

it?

2. How does the Boost FV programme take place in your class?

a) How does the FV get to the class room?

b) How is the FV stored and does it stay fresh?

c) Is the FV being cut up and by whom?

d) When is the FV eating taking place and have you allocated a specific time for it (a

break)?

e) Who is in charge of cleaning up afterwards?

f) How much time do you spend on the FV programme?

3. How is the eating environment?

a) In your opinion, has a pleasant eating environment been created in your class?

b) Do you use the components of the Boost class kit e.g. candles, cutting tools and lemon juice?

c) Have you talked with the pupils about the different FV delivered (e.g. taste, country of origin)?

d) Have a sense of community among pupils evolved around the FV programme?

e) Have the pupils begun eating a larger variety of FV after the introduction of the FV

programme?

4. What do you think about having the Boost FV programme in your class?

a) Does it take time from teaching?

b) Do you experience the FV programme as a burden?

c) Does the FV programme result in many quarrels or a better atmosphere in class?

5. How do you experience the support from you head master concerning project participation?

6. How do you experience the support to your participation in the project from your colleagues?

7. If you were to have a FV programme in the future, what should be different?
