# Supplementary material for: Implementing a free school-based fruit and vegetable programme: barriers and facilitators experienced by pupils, teachers and produce suppliers in the Boost study
Source: BMC Public Health. 2014 Feb 11;14:146. doi: 10.1186/1471-2458-14-146 (PMC3946026; doi:10.1186/1471-2458-14-146)
Supplement: Additional file 3 — Observation guide for the Boost fruit and vegetables (FV) break. [file 1471-2458-14-146-S3.docx]

**Additional file 3. Observation guide for the Boost fruit and vegetables (FV) break**

1. Before the Boost FV break

a) How is the atmosphere in class immediately before the pupils eat FV?

2. During the FV break

a) How is the FV eating initiated (e.g. the teacher decide that it is time for a FV break, pupils suggest it)?

b) When is the FV eaten?

c) Who is bringing the FV to class and from where?

d) Who prepares the FV, how and where?

e) How is the atmosphere in class when pupils get the FV?

f) Is the FV being distributed to the pupils at their tables or is it picked up by each pupil?

g) Who is eating the FV (girls/boys)?

h) How is the teacher reacting to the FV eating?

i) How is the appearance of the FV delivered?

j) What is going on while the pupils eat the FV?

k) How much time is spent on the FV eating?

l) Who is cleaning up after the pupils have eaten the FV?

m) On average, how much time is spent on the FV break from initiation until it has been cleaned up?

3. After the FV break

a) How is the atmosphere in class immediately after the pupils have eaten FV?
