# Supplementary material for: Implementing a free school-based fruit and vegetable programme: barriers and facilitators experienced by pupils, teachers and produce suppliers in the Boost study
Source: BMC Public Health. 2014 Feb 11;14:146. doi: 10.1186/1471-2458-14-146 (PMC3946026; doi:10.1186/1471-2458-14-146)
Supplement: Additional file 4 — Topic areas for interviews with fruit and vegetable (FV) suppliers on the Boost FV programme. [file 1471-2458-14-146-S4.docx]

**Additional file 4. Topic areas for interviews with fruit and vegetable (FV) suppliers on the Boost FV programme**

1. Who is involved in the Boost FV delivery in your store?

2. Does everybody in the store know about the Boost project and the tasks associated with it?

3. How big a workload has it been for you to participate in the project?

4. How has the delivery form of getting the FV to the schools worked for you?

5. Have you been able to deliver the FV ordered?

6. How has the order form (provided by the Boost project group) worked for you?

7. Have you received sufficient information concerning the implications of participating in the Boost project?

8. How has the contact/cooperation been with the school?

9. Have you experienced any problems or challenges in relation to your participation in the project?

10. Have there been any advantages or disadvantages of participating in the project?

11. What were the main reasons that motivated you to participate in the project?

12. Could you imagine yourself participating in schools FV programmes in the future or should something be changed in the organization of these programmes for you to participate again?
